# Neuroprotective Activity of 3‑((6-(Phenylethynyl)pyridin-3-yl)oxy)quinuclidine: A Potential Ligand for the Treatment of Alzheimer’s Disease

**DOI:** 10.1021/acschemneuro.5c00527

**Published:** 2025-11-13

**Authors:** Pablo S. Cavagnero, Yaíma Sánchez, Brian Fell, Oscar Ramírez Molina, Javiera Gavilán, Efraín A. Polo, Jorge Fuentealba, Margarita Gutierrez, Claudio A. Jiménez, Jhon J. López

**Affiliations:** † Universidad de Concepción, Facultad de Ciencias Químicas, Departamento de Química Orgánica, Concepción 4130000, Chile; ‡ 28066 Universidad de Talca, Instituto de Química de Recursos Naturales, Talca 3460000, Chile; § Universidad de Concepción, Facultad de Ciencias Biológicas, Departamento de Fisiología, Concepción 4130000, Chile; ∥ 28033 Pontificia Universidad Católica de Chile, Facultad de Química y de Farmacia, Departamento de Química Orgánica, Santiago 7820436, Chile

**Keywords:** Alzheimer’s disease, β-amyloid, cytotoxicity, neuroprotection, Aβ aggregation

## Abstract

Alzheimer’s disease (AD) is a neurodegenerative disorder marked by the accumulation of β-amyloid (Aβ) peptides, which disrupt neuronal homeostasis through their neurotoxic effects. Aβ aggregates interfere with synaptic function by interacting with nicotinic acetylcholine receptors (nAChRs), particularly the α7 subtype, thereby impairing cholinergic signaling, which is crucial for cognition and memory. Pharmacological advancements have identified Positive Allosteric Modulators (PAMs) as promising therapeutic agents to counteract Aβ neurotoxicity. PAMs enhance nAChR activity by binding to allosteric sites, thereby reducing Aβ-induced neurotoxicity without competing with acetylcholine. This study evaluates 3-((6-(phenylethynyl)­pyridine-3-yl)­oxy)­quinuclidine (**EQ-04**), a novel PAM with high selectivity for the α7 nAChR subtype, demonstrating neuroprotective potential. *In vitro* analyses using PC-12 cells evaluated the cytotoxic and neuroprotective properties of **EQ-04**. Cytotoxicity assays confirmed **EQ-04**’s safety, showing no adverse effects on cell viability across concentrations. **EQ-04** significantly enhanced cell viability by 37% at 1 nM against Aβ toxicity and inhibited Aβ aggregation. These findings highlight the potential of **EQ-04** as a neuroprotective agent for Alzheimer’s disease (AD) therapy, warranting further investigation into its pharmacokinetics and *in vivo* efficacy.

## Graphical abstract

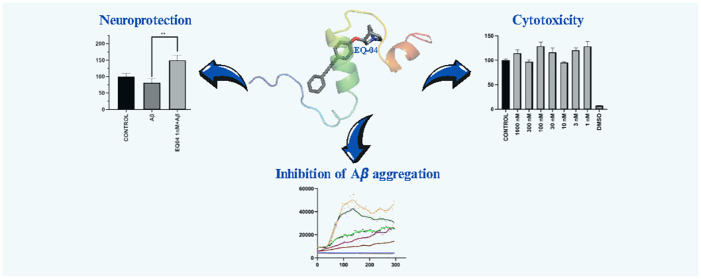

## Introduction

Alzheimer’s disease (AD) is a progressive neurodegenerative pathology characterized by memory loss, multiple cognitive impairments, personality changes, and behavioral changes.^1,2^ To date, there is no drug capable of stopping, reversing, or preventing the neurodegenerative process of AD.^3^ One of the main characteristics of AD is the accumulation of the β-amyloid peptide (Aβ),^1^ characterized by the binding of several Aβ monomers, which form insoluble fibrils responsible for the neurotoxicity.^4^ Interestingly, several studies have shown that this peptide binds with high affinity to nicotinic acetylcholine receptors (nAChRs), specifically the α7 subtype, thereby affecting their normal functioning.^5–7^ Although this interaction is not entirely clear, it has been suggested that α7 nAChR is involved in the toxicity of Aβ and the pathogenesis of AD.^8^

The α7 subtype is widely distributed in the central nervous system (CNS) in regions involved in cognitive functions and memory, including the hippocampus, cortex, and subcortical limbic areas, where it contributes to cognition, sensory processing, information, attention, working memory, and reward pathways.^9,10^ Another characteristic of α7 is that it also acts as a metabotropic receptor, triggering several signal transduction pathways, as well as the release of calcium (Ca^2+^) from intracellular stores.^10–14^ This metabotropic activity has been associated with synaptic plasticity and neuroprotection, including against Aβ.^15–17^ For this reason, increased expression of α7 might be beneficial for the CNS because moderate activation of these receptors could increase cellular resistance to brain damage, which has been demonstrated *in vivo* experiments and *ex vivo* models of dementia, brain ischemia, and traumatic brain damage.^9^

Therapeutic strategies for correcting cholinergic deficits included increasing acetylcholine (ACh) synthesis, stimulation of cholinergic receptors, and inhibition of acetylcholinesterase (AChE) and butyrylcholinesterase (BChE) enzymes^18^ However, increased synthesis of ACh has shown little efficacy, and the use of cholinergic agonists induces receptor desensitization, leading to tolerance and, therefore, loss of beneficial effects. At the same time, inhibiting acetylcholinesterase only produces short-term improvements in symptoms but does not prevent disease progression.^19^

A new strategy proposed to improve cholinergic function involves the use of Positive Allosteric Modulators (PAMs). These compounds bind to the receptor at a different site (allosteric site) than the agonist binding site (orthosteric site).^10,19–22^ PAMs are compounds that do not induce any response in the receptor. However, in the presence of the orthosteric agonist ACh, they can increase the response.^9,19–23^ Furthermore, PAMs prevent overstimulation of the receptors and act as neural protectors. Choline is an endogenous selective agonist of the α7 nAChRs. Cerebrospinal fluid (CSF) contains choline at concentrations much lower (∼5–10 μM) than its EC_50_ (∼0.5–1.5 mM). Moreover, choline exhibits a much greater potency for desensitization (IC_50_ ∼ 40 μM) to activate α7 nAChRs. Cell death is a significant source of choline caused by phosphatidylcholine breakdown, the principal plasma membrane phospholipid, into choline and diacylglycerol. Given the low ambient concentrations of choline in the CSF under physiological conditions, it is unlikely that in the absence of cholinergic synaptic inputs or exogenous nicotinic agents, native α7 nAChRs are persistently activated by endogenous choline. Therefore, in the presence of PAMs, endogenous choline may become effective in producing moderate persistent activation of α7 nAChRs and the corresponding elevation in the Ca^2+^ influx and neuronal excitability, supporting neuroprotection and cognition.^24^ However, the presence of a PAM may not require the involvement of an agonist to mediate a local neuroprotective effect.^10,14,19,25,26^ Studies have shown that it improves learning and reverses memory loss and some sensory deficits typical of psychiatric diseases.^27^ An example is BNC375 (Figure 1A), a PAM of α7 which demonstrates robust procognitive effects in rodent and nonhuman primate models.^28,29^ Overall, these findings suggest that α7 nAChR PAMs have therapeutic potential in central nervous system disorders characterized by cognitive deficits.^28,29^

**Figure 1:**
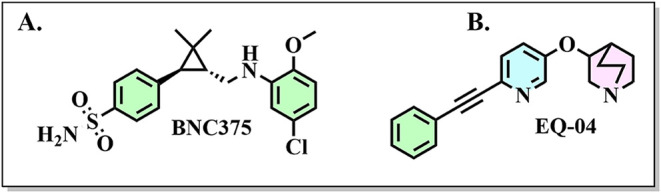
Representative examples of PAMs selective to human α7 nAChR. A. Chemical structure of BNC375. B. Chemical structure of EQ-04.

Recently, we discovered a new PAM 3-((6-(phenylethynyl)­pyridine-3-yl)­oxy)­quinuclidine (**EQ-04**), selective for α7 nAChR (Figure 1B). Electrophysiological recordings in *Xenopus laevis* oocytes showed that **EQ-04** is positive modulator of α7 (EC_50_ = 12.6 ± 3.32 μM) with maximal potentiation of ACh response EC_20_ (*E*_max_ = 850 ± 120%).^30^

Moreover, computational studies revealed the preference of **EQ-04** for an intersubunit site in the transmembrane domain and highlighted some putative vital interactions.^30^

This study aimed to explore the neuroprotective activity of **EQ-04** against Aβ toxicity. We performed cytotoxicity and neuroprotection studies in cells and neurons exposed to Aβ. Also, molecular docking studies were conducted. Our results showed that 1nM **EQ-04** inhibits Aβ toxicity in PC-12 cells and neurons. Furthermore, it inhibited the aggregation rate of Aβ.

## Results and Discussion

### Evaluation of the Cytotoxicity of **EQ-04**

Before performing the neuroprotective studies, it was necessary to establish the toxic effects of the compound. For this purpose, PC-12 cells were incubated for 24 h with different concentrations of **EQ-04** (1000, 300, 100, 30, 10, 3, and 1 nM, respectively), and cell viability was assessed.^3,31–34^

In the MTT experiments, we observed that none of the tested compound concentrations (1–1000 nM) proved to be cytotoxic. No statistically significant changes in cell viability were detected compared to the untreated control group (Figure 2A).

**Figure 2:**
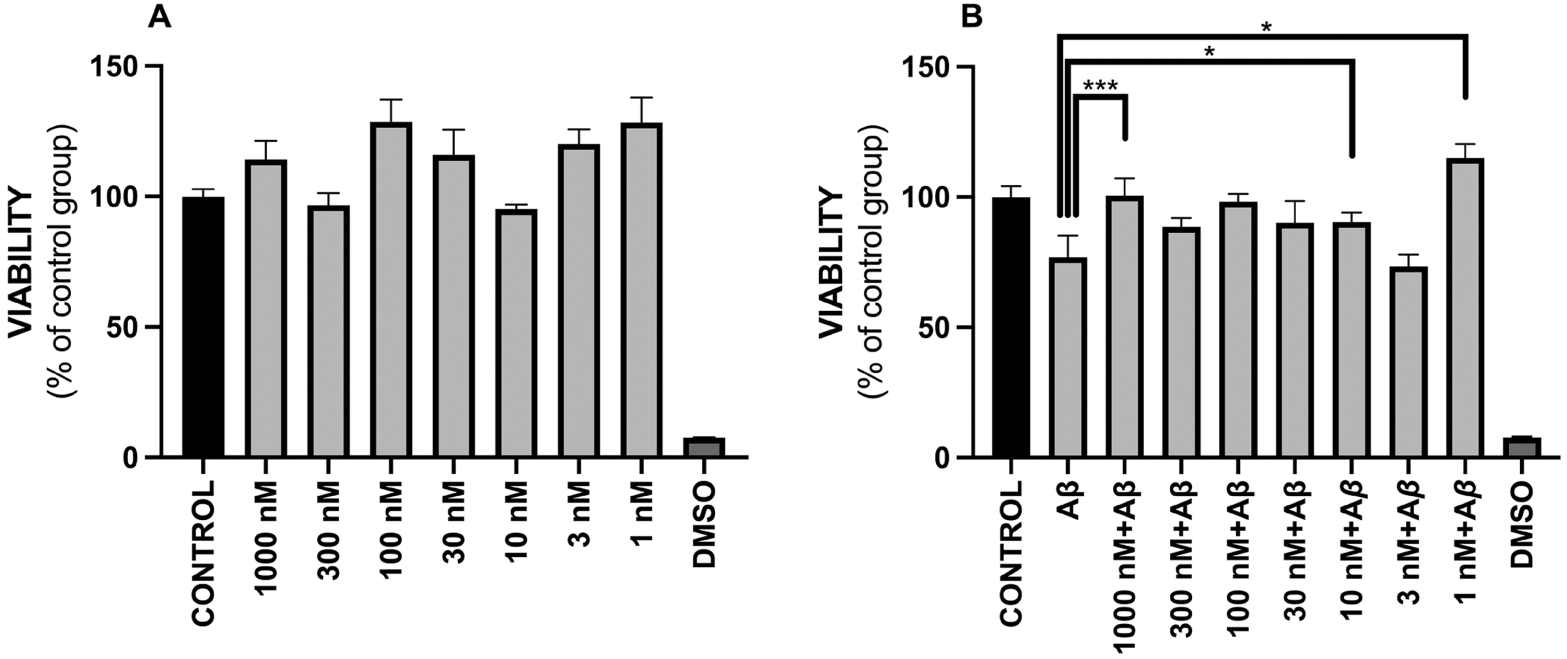
(A) Effect of **EQ-04** on cellular viability of PC-12 cells exposed to a wide interval of concentrations (1–1000 nM) of the compound for 24 h. DMSO (50%) was used as the positive control for toxicity (*n* = 3). (B) Cytoprotective effects of **EQ-04** on the viability of PC-12 cells exposed to a wide range of concentrations (1–1000 nM) of the compound for 24 h and Aβ at 0.5 μM. DMSO at 50% was used as a positive control of toxicity (*n* = 3). Data is shown as ± SEM *p < 0.05 and ***p < 0.001 vs Aβ.

### Evaluation of the Cytoprotective Effect of **EQ-04** against Aβ Toxicity

To assess the potential protective effect of **EQ-04** against the toxicity associated with Aβ, we used the same methodologic approach described in the previous section to evaluate the cytoprotective effect of **EQ-04** on PC-12 cells treated chronically with Aβ (0.5 μM for 24 h), using the same range of concentrations previously evaluated, where it was observed that at concentrations of 1000, 10 and 1 nM a statistically significant recovery of viability was achieved compared to the positive control group treated only with Aβ. Notably, while no statistically significant recovery of cell viability was observed, the remaining concentrations exhibited increases in viability compared with the Aβ positive control (Figure 2B).

All viability values reported at the different concentrations were statistically like those of the untreated control group. The best viability recovery value was observed at a concentration of 1 nM (viability recovery of approximately 37% was observed). Therefore, we decided to investigate the potential neuroprotective effects of our compound *in vitro* models of Alzheimer’s disease (AD).

### Evaluation of the Neuroprotective Activity in Hippocampal Neurons

Considering that α7 nAChR activation protects against Aβ-induced cytotoxicity in AD,^35–38^ we aimed to evaluate **EQ-04** in a neuronal model exposed to Aβ. To assess the effects on synaptic function, labeling was performed with an antibody against Synaptic Vesicle Protein 2 (SV-2), a glycoprotein located in synaptic vesicles that undergo Ca^2+^ regulated exocytosis, which is essential for the normal functioning of the nervous system. Several studies have shown that phenotypes associated with mutations in SV-2 genes indicate that this protein is vital for normal synaptic function.^37,38^ Changes in immunoreactivity were assessed by fluorescence microscopy. Labeling of the mitochondrial protein mitofusin-1, which is one of the essential proteins in mitochondrial dynamics, specifically for mitochondrial fusion and is indicative of mitochondrial health, was also performed.^39–43^ The two proteins were labeled with primary antibodies, which in turn were labeled with the corresponding secondary antibodies: for SV-2, the secondary antibody emitted green, while for mitofusin-1 (mitochondrial dynamics), it emitted red. The measurements correspond to the fluorescence intensity. All values were normalized to those of the control group, which were considered 100%.

To evaluate the neuroprotective activity of **EQ-04** and based on the promising results obtained in cells, we decided to treat neurons obtained from mouse embryonic hippocampus with Aβ peptide and with **EQ-04**, using concentrations of 1 and 1000 nM, respectively (the most effective in neuroprotective assays). We used a negative control group (untreated neurons; Figure 3A), a positive control of neurons exposed only to Aβ peptide (Figure 3D), and two groups treated only with **EQ-04** at the two selected concentrations (Figure 3B,E). These assays showed that incubation with the compound **EQ-04** at the two concentrations tested induced a marked increase in the fluorescence emitted by SV-2 in the neurons treated with the compound alone (Figures 3B,E, and 4A).

**Figure 3:**
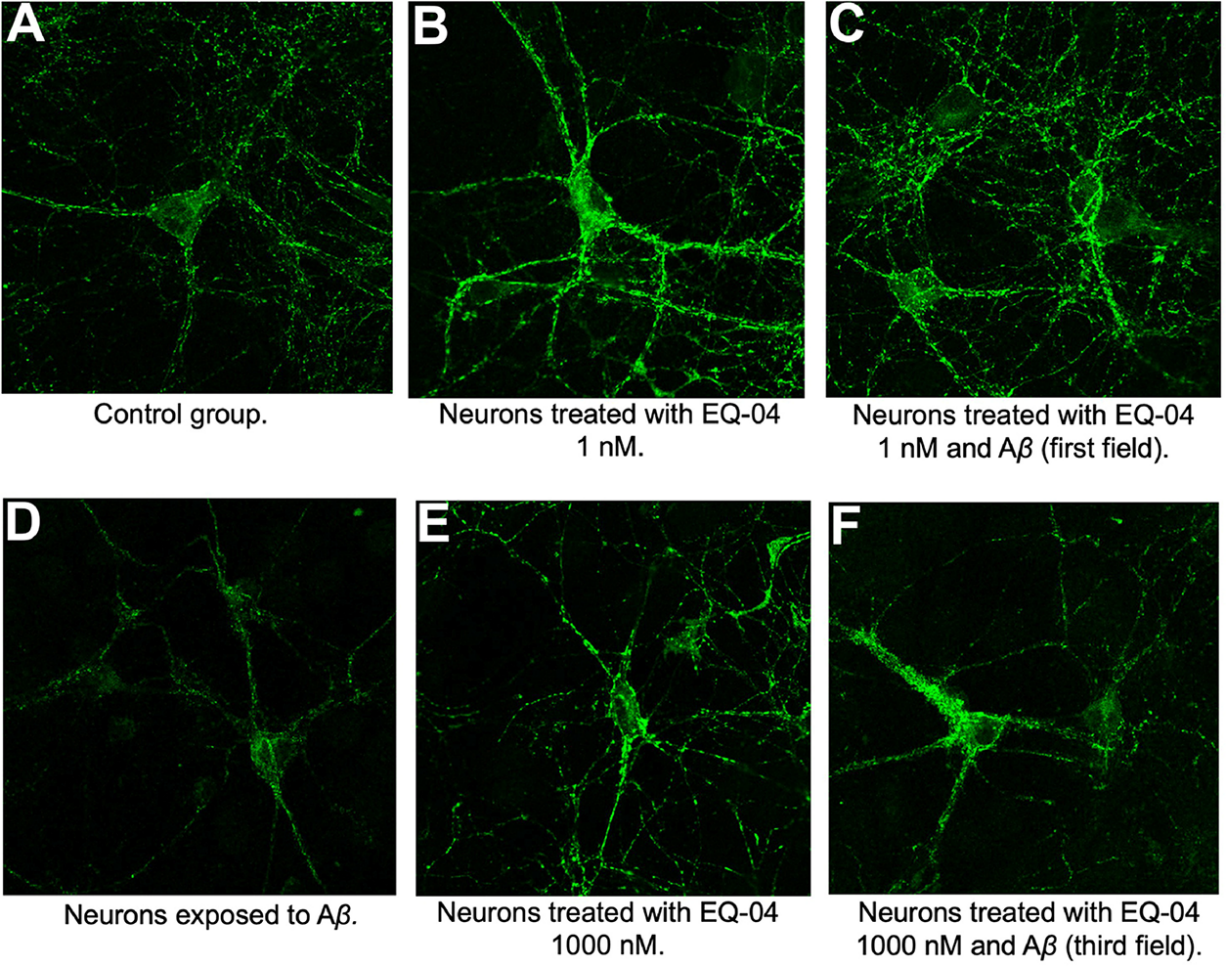
Changes in the expression levels of SV2 (green fluorescence) induced by **EQ-04** and Aβ in primary cultures of rat hippocampal neurons. Representative images obtained by confocal microscopy of hippocampal neurons at 10 days *in vitro* (10 DIV) treated during 24 h with Aβ (0.5 μM (D)), **EQ-04** (1 and 1000 nM (B, E), respectively), and Aβ + **EQ-04** (1 nM and 1000 nM (C, F), respectively). The control group (untreated neurons (A)) is shown.

**Figure 4:**
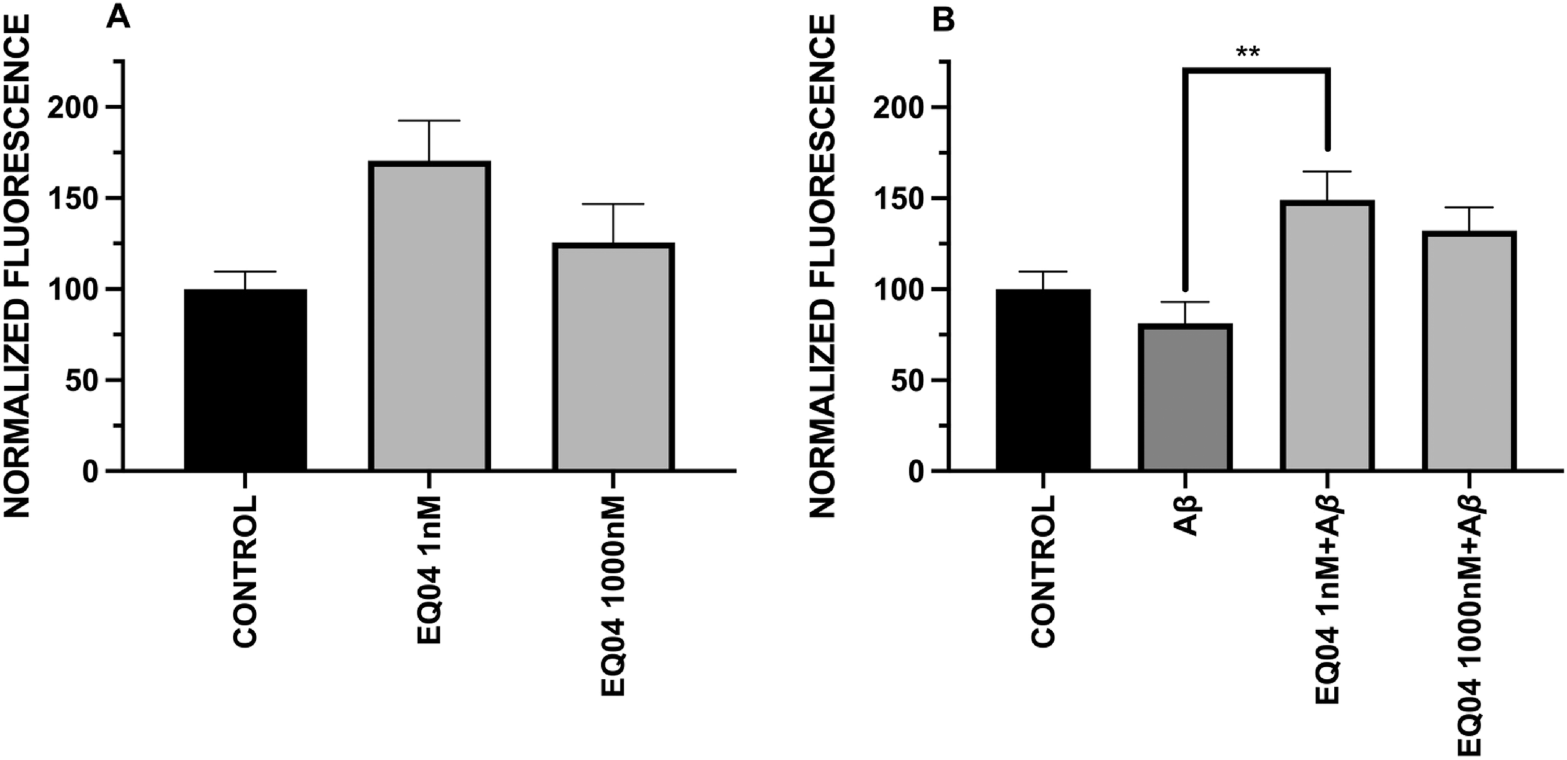
(A) Effect of the incubation with **EQ-04** in the expression of the presynaptic glycoprotein SV-2. (B) Effect of the coincubation with **EQ-04** (1 nM and 1000 nM) and Aβ (0.5 μM) on the expression of the presynaptic glycoprotein SV-2 to protect neurons from the synaptic failure associated with Aβ peptide. Data is shown as ± SEM **p < 0.01 vs Aβ.

In contrast, exposure of neurons to Aβ alone caused a 19% decrease in fluorescence compared to the control (Figure 3D). Simultaneously, coincubation with the peptide and **EQ-04** at study concentrations showed a significant increase in SV-2 levels (Figure 3C,F), where the 1 nM concentration generated a log-normalized fluorescence of 149%, higher than that observed in the control (Figure 4B). This result demonstrates the protective effect of **EQ-04** against the well-established toxicity of the Aβ peptide, as observed in PC-12 cells, and is now confirmed in hippocampal neurons. This compound has been shown to protect the neuronal network from neurotoxic and, eventually, neurodegenerative stimuli, such as Aβ.

It is vital to consider that given the excellent results obtained with the 1 and 1000 nM concentrations, which are also in agreement with the results previously obtained for the cytoprotection for PC-12 cells, we believe that *in vivo* studies should be carried out with this compound in the range of concentrations evaluated (1–1000 nM).

Regarding the behavior of mitofusin-1 (see Supporting Information), no significant changes were observed in the control or positive control group (treated only with the peptide; see Figure S2 in Supporting Information). However, we consider that the most relevant result was obtained regarding the increase in the amount of presynaptic protein.

### Aβ Aggregation Assays

Aggregation is a process characterized by the interaction of Aβ monomers, which form soluble oligomers (dimers, trimers, etc.) that are highly toxic to neurons; these can further join together to produce insoluble amyloid fibrils that tend to accumulate in the brain as senile plaques typical of AD.^44^ Due to the neuroprotective properties shown by **EQ-04**, we decided to perform aggregation assays of Aβ monomer using 1 and 10 μM concentrations of the compound.

The results indicated that coincubation of 1 μM **EQ-04** with Aβ monomers (Figure 5, yellow line) did not prevent the aggregation process, since the fluorescence kinetics were almost identical to those of the control condition (AβOs + THT, green line). The apparent increase in fluorescence at this concentration is therefore unlikely to represent an inhibitory effect. At low concentrations, it is possible that the conformation of Aβ or the binding of THT may be altered, allowing the exposure of additional binding sites and generating an increase in fluorescence, even if fibrillar aggregation has not increased. Some compounds have been shown to promote the formation of early oligomers, which can expose more binding sites for THT and increase the signal, even if mature fibers are not formed.^45^ A similar phenomenon was previously described for chalcone derivatives reported by our group, which also displayed intrinsic fluorescence in the presence of THT.^3^

**Figure 5:**
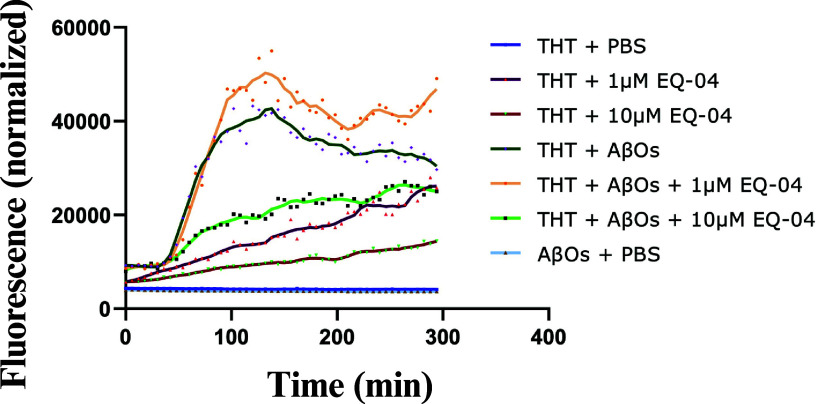
Fluorescence curve with and without **EQ-04**. THT + PBS represents control conditions (blue line). THT + 1 μM **EQ-04** without Aβ (purple line). THT + 10 μM **EQ-04** without Aβ (brown line). THT + Aβ without **EQ-04** (dark green line). THT + Aβ + 1 μM **EQ-04** (yellow line). THT + Aβ + 10 μM **EQ-04** (light green line). Aβ + PBS low basal fluorescence, as would be expected in the absence of THT to detect β-amyloid structures (light blue line).

In contrast, when Aβ monomers were incubated with 10 μM **EQ-04** (Figure 5, light green line), a marked decrease in the fluorescence signal was observed compared with the control condition. This reduction suggests effective inhibition of the fibril elongation process, reducing both aggregation kinetics and THT fluorescence, consistent with previously reported studies.^46^ Taken together, these findings demonstrate that the inhibitory effect of **EQ-04** on Aβ aggregation becomes evident only at concentrations ≥ 10 μM, while lower concentrations may contribute to fluorescence artifacts without significantly affecting the aggregation kinetics.

### Molecular Coupling Studies between **EQ-04** and Aβ

To understand the inhibition assays of Aβ monomer aggregation, molecular docking studies were performed on the compound **EQ-04** using the crystal structure of the Aβ_1–40_ monomer (PDB code1AML).^47^ In this monomer, three regions have been described for the binding of various types of compounds: **1**. A hydrophobic region (LEU-17–ALA-21). **2**. A *Loop* (ASP-23-LYS-28). **3**. A second hydrophobic region (GLY-29-MET-35).^48^ Based on the above, we decided to perform molecular docking of **EQ-04** in the three regions of the monomer to determine its binding preference (see Figure 6A–C). Structural and binding energy (Δ*G*_bind_) analyses estimated for each of the complexes (ligand-protein) in regions I (Figure 6A), II (Figure 6B) and III (Figure 6C) showed favorable values ranging of −4.19, −2.97 and −2.57 kcal/mol, respectively. Also, inhibition constants (*K_i_*) were estimated for **EQ-04** in the three regions, where we obtained values of 1.09 μM, 6.67 μM, and 13.04 μM, respectively, for the interaction with the monomer (Figure 6A–C).

**Figure 6:**
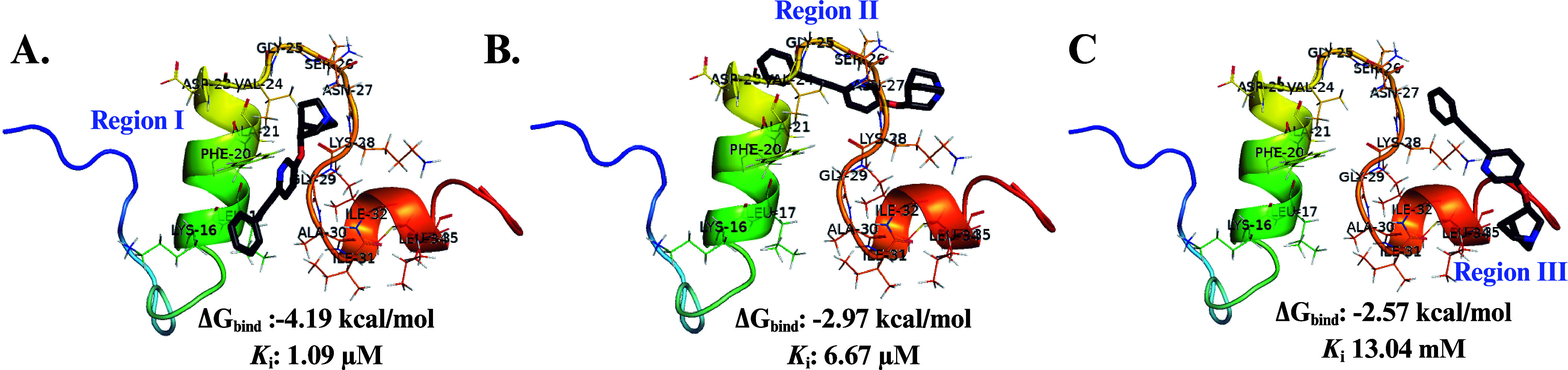
Molecular docking studies of the EQ-04 ligand (in gray) with the three regions of the 1AML crystal structure (colored).^47^ (A) Molecular interaction of **EQ-04** with amino acids (represented in line) of region I (LEU-17-ALA-21). (B) Molecular interaction of **EQ-04** with amino acids (represented in line) of region II (ASP-23-LYS-28). (C) Molecular interaction of **EQ-04** with amino acids (represented in line) of region III (GLY-29-MET-35). Binding energies (Δ*G*_bind_) and inhibition constants (*K_i_*) for each complex were obtained from AutodockTools 4.2.

Our results suggest that **EQ-04** would form very favorable (−4.19) and hydrophobic interactions in region I (Figure 6A). Where the aromatic ring of the phenylacetylene group would form π-π stacked interaction with PHE-20, π-alkyl interactions with LEU-17, and ALA-30. On the other hand, the pyridine ring would be forming π-alkyl interactions with ILE-32, ALA-21, and VAL-24, while the quinuclidine ring would only form alkyl interactions with VAL-24 and ALA-21­(Figure 7). These interactions would be key to the stabilization of the ligand-protein complex, thereby preventing the binding of Aβ monomers and the generation of toxic oligomers or peptide-like aggregates, which would explain the results obtained in the aggregation assays at a concentration of 10 μM of **EQ-04**.

**Figure 7:**
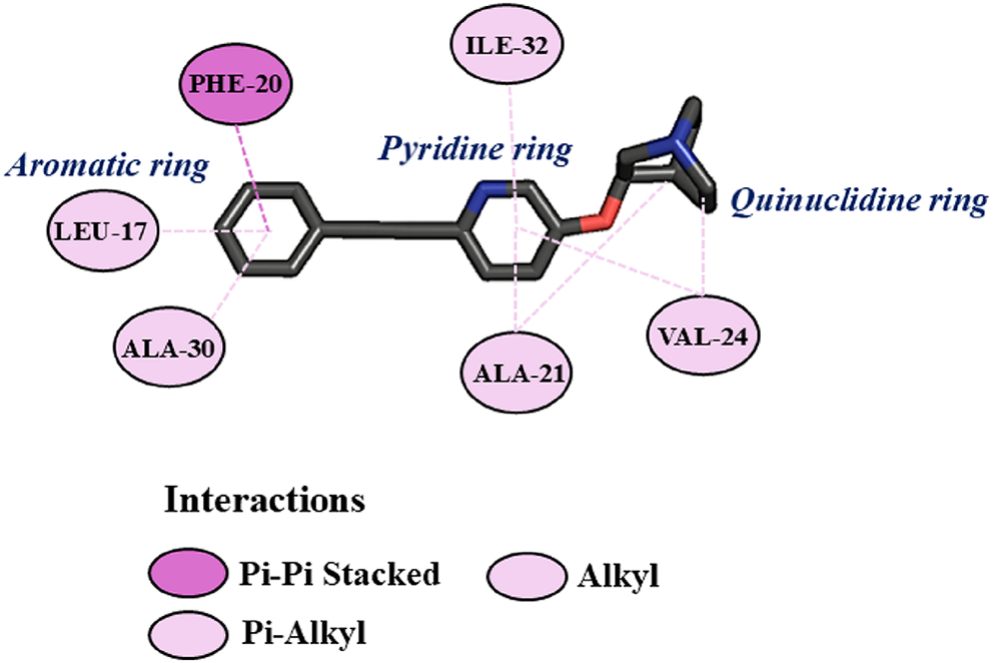
2D diagram for the **EQ-04**—Aβ complex, which illustrates the main molecular interactions in region I of the Aβ monomer.

### Inhibition Assays against AChE and BChE

The inhibition of these enzymes has been considered a potential target for treating Alzheimer’s disease (AD), as compounds with inhibitory activity against these enzymes can maintain adequate levels of acetylcholine (ACh), thereby allowing for the restoration of cognitive functions.^49–51^ We performed inhibition assays against AChE and BChE, which are present in nerve tissues. The results showed that **EQ-04** had no inhibitory activity compared to galantamine, used as a reference (see Supporting Information).

## Conclusions

This study aimed to explore the neuroprotective activity of **EQ-04** PAM against the toxicity of Aβ. In this study, we evaluated the cytotoxicity and neuroprotective effects of **EQ-04**. We also assessed the ability of this compound to inhibit the enzymatic activity of AChE and BChE. We were able to establish that none of the studied concentrations were toxic to PC-12 cells. Moreover, at a concentration of 1 nM, the compound showed a neuroprotective effect against Aβ toxicity, exhibiting a significant increase in cell viability, as well as an excellent neuroprotective effect against Aβ in mouse hippocampal neurons. Moreover, **EQ-04** inhibited the aggregation kinetics of the Aβ peptide, suggesting a more specific action on the primary molecule responsible for AD. In addition, molecular docking studies showed that **EQ-04** would generate energetically favorable interactions in different binding regions of the Aβ peptide. However, it showed no inhibitory activity against AChE and BChE, suggesting that the principal pathway of the compound is against cholinergic failure, mediated by α7 nAChR potentiation and inhibited the aggregation kinetics of the Aβ peptide.

Our results provide consistent evidence that **EQ-0**4 attenuates Aβ aggregation, reduces neuronal toxicity, and improves cell viability, thus supporting its role as a promising neuroprotective candidate. While these findings strongly suggest beneficial activity, we recognize that confirmation of the neuroprotective profile will ultimately require complementary *in vivo* studies, including animal models, oxidative stress measurements, and histopathological analyses. Therefore, the current work should be considered as a mechanistic foundation that paves the way for future translational investigations of **EQ-04**. In addition, the relatively simple structure of this compound may be the starting point for generating new, improved derivatives.

## Materials and Methods

### Chemical Synthesis

**EQ-04** was synthesized using the same methodology described previously (see Supporting Information).^30^

### Preparation of **EQ-04** for Biological Assays

**EQ-04** was dissolved to achieve a final concentration of 100 μmol/L in 10 mL of dimethyl sulfoxide (DMSO) creating a stock solution. For subsequent assays, this stock solution was serially diluted three times in Dulbecco’s phosphate-buffered saline (DPBS) to prepare solutions with concentrations of 10,000, 1000, and 100 nM. These were further diluted to achieve final experimental concentrations of 1000 nM, 300 nM, 100 nM, 30 nM, 10 nM, 3 nM, and 1 nM.

### PC-12 Cells

PC-12 cells were cultured in DMEM medium supplemented with 5% fetal bovine serum, 5% horse serum, and 1% penicillin-streptomycin. The cells were maintained in a temperature-controlled incubator at 37 °C with 5% CO_2_ and used for experiments once they reached approximately 80% confluency.

### Aggregation of Aβ_1–40_ Peptide and Obtention of Oligomers

The Aβ_1–40_ peptide (Peptide, Bogart, GA, USA) was initially dissolved in DMSO at a concentration of 2.3 mM. A 2 μL aliquot of this stock solution was further diluted in sterile distilled water to a concentration of 80 μM. This solution was stirred at 500 rpm using a magnetic stirrer for 2 h at room temperature to promote oligomer formation. The peptide was then used at a working concentration of 0.5 μM.

### Thioflavin T Binding Assay

Aβ aggregation was performed on a 96-well plate in the presence or absence of the compounds in DPBS buffer with 20 μM of Thioflavin T (THT, Sigma Aldrich). The aggregation process was followed by fluorescence measurements of the Thioflavin T-Aβ complex (ex: 440 nm, em: 485 nm) every 3 min for 4 h. The plate was kept at room temperature with an orbital agitation of 500 rpm.

### Cell Viability Assay

Various concentrations of **EQ-04** were incubated with PC-12 cells in 96-well microplates for 24 h. The medium was extracted and then replaced with 100 μL of thiazolyl blue tetrazolium bromide (MTT) 1 mg/mL. The resulting formazan crystals were dissolved in isopropyl alcohol and incubated at 37 °C for 15 min. Absorbance was measured using a NOVOstar microplate reader (BMG, Germany) at wavelengths of 560 and 620 nm, and the data were analyzed using NovoStar Software to quantify differences under the various experimental conditions.

### Evaluation of the Neuroprotective Activity

Neuroprotective activity was assessed using immunofluorescence techniques on hippocampal embryonic neurons derived from C57BL/6 mice (E18).^52,53^

### Hippocampal Cultures

Primary hippocampal neurons from 18-day-old embryonic rats were cultured at a density of 300,000 cells/mL on poly-l-lysine-coated plates. The cultures were maintained in minimal essential media (MEM) supplemented with 10% horse serum, 4 μg/mL DNase, and 2 mM l-glutamine for the first 24 h. The medium was then replaced with MEM containing 2% horse serum, 2% fetal bovine serum, and 0.5% N_2_ supplement. Neurons were incubated in a temperature-controlled environment at 37 °C with 5% CO_2_ and used after 9 days *in vitro* (DIV).^52,53^

### Immunofluorescence

Hippocampal neurons at 10 DIV were treated for 24 h with soluble oligomers of Aβ_1–40_ (0.5 μM) and **EQ-04** at 1 nM and 1000 nM concentrations. After treatment, samples were washed with PBS 1× and fixed with paraformaldehyde in PBS for 15 min at room temperature. Nonspecific binding was blocked using 10% horse serum, and cells were permeabilized with 0.1% Triton X-100 for 20 min. Specific antibodies were used to detect synaptic vesicle glycoprotein 2 (SV2) and mitofusin-1. Cells were mounted using DAKO Fluorescent Medium, and images were captured using a LEICA SP-8 spectral confocal microscope at the Center for Advanced Microscopy, University of Concepcion. Image analysis was conducted using the MacBiophotonics ImageJ software. To quantify mitofusin-1 levels, three random regions of interest (ROIs) were selected from the neuronal soma, and SV2 expression was monitored by comparing the fluorescence exhibited at the primary processes of the neurons.^52,53^

### Statistical Analysis

Results are plotted as the mean ± SEM and expressed as a percentage of the nontreated control group. Analyses were performed using a one-way ANOVA, and significant statistical values were *p < 0.05, **p < 0.01, and ***p < 0.001 versus control; and #*p* < 0.05, ##*p* < 0.01, and ###*p* < 0.001 versus SO-Aβ. All analyses were conducted using GraphPad Prism software (GraphPad Prism, CA, USA).

### Molecular Docking

To study characteristics of the principal protein–ligand interactions, molecular docking of the Aβ_1–40_ was done using the AutoDock 4.2 software suite.^54^ In general terms, grid maps were calculated using the AutoGrid option and centered on the binding sites. The volumes chosen for the grid maps were made up of 40 × 40 × 40 points, with a grid-point spacing of 0.375 Å. For three regions, a grid covering the whole region was used instead. The AutoTors option of the AutoDock 4.2 software suite was used to define the rotating bonds in the ligand. In the Lamarckian genetic algorithm dockings,^55^ individuals were selected from a population of 1500, with a maximum of 2.5 × 10^6^ energy evaluations, 27,000 generations, a mutation rate of 0.02, and a crossover rate of 0.80. The docked compound complexes were built using the lowest docked-energy binding positions. The Aβ_1–40_ crystal structure (PDB code 1AML)^47^ was retrieved from the RCSB Protein Data Bank,^56^ and the nonprotein atoms were removed using PyMOL software.^57^ The structure of **EQ-04** was generated using ChemDraw (Perkin-Elmer Informatics) and subsequently optimized with Spartan18. Polar hydrogens and charges were added to the protein and ligand structures with the AutoDock Tools^54^ suite before docking. The analysis of the molecular interactions of the **EQ-04** in the three regions of the Aβ_1–40_ was carried out using the software BIOVIA,^58^ and the figures were created with PyMOL.

## Supplementary Material



## Data Availability

The compound **EQ-04** is available from J.J.L.

## Notes

Confocal microscopy images of the mitofusin 1 protein; effect of the incubation with **EQ-04** in the expression of the mitochondrial protein mitofusin 1; evaluation of the potential effect as inhibitors of the enzyme acetylcholinesterase; IC_50_ values for AChE and BChE for **EQ-04** and galantamine; general procedure for preparation of **EQ-04** (PDF)

## Abbreviations

ACh: acetylcholine
AChE: acetylcholinesterase enzyme
BChE: butyrylcholinesterase enzyme
AD: Alzheimer’s disease
**A** ***β***: β-amyloid peptide
DMEM: Dulbecco’s Modified Eagle Medium
DMSO: dimethylsulfoxide
**EC**_**50**_: ligand concentration producing half-maximal potentiation
**IC**_**50**_: ligand concentration producing half inhibition
PAM: positive allosteric modulator
mM: mmol/L
nM: nmol/L
μM: μmol/L
SEM: standard error of the mean
THT: thioflavine
