# Supplementary material for: Neuroprotective Activity of 3‑((6-(Phenylethynyl)pyridin-3-yl)oxy)quinuclidine: A Potential Ligand for the Treatment of Alzheimer’s Disease
Source: ACS Chem Neurosci. 2025 Nov 13;16(23):4502–10. doi: 10.1021/acschemneuro.5c00527 (PMC12679543; doi:10.1021/acschemneuro.5c00527)
Supplement: Supplementary file 1 [file cn5c00527_si_001.pdf]

Supporting Information

Neuroprotective Activity of 3-((6-(Phenylethynyl)pyridin-3-yl)oxy)quinuclidine. A Potential Ligand for the Treatment of Alzheimer's Disease

Pablo S. Cavagnero,[†] Yaíma Sánchez,[‡] Brian Fell,[†] Oscar Ramírez Molina,[§] Javiera Gavilán,[§] Efraín A. Polo,[†] Jorge Fuentealba,[§] Margarita Gutierrez,[‡] Claudio A. Jiménez[*,†] and Jhon J. López[*,‖]

[†]Universidad de Concepción, Facultad de Ciencias Químicas, Departamento de Química Orgánica, Concepción 4130000, Chile

[‡]Universidad de Talca, Instituto de Química de Recursos Naturales, Talca 3460000, Chile

[§]Universidad de Concepción, Facultad de Ciencias Biológicas, Departamento de Fisiología, Concepción 4130000, Chile

[‖]Pontificia Universidad Católica de Chile, Facultad de Química y de Farmacia, Departamento de Química Orgánica, Santiago 7820436, Chile

*Corresponding author: cjimenez@udec.cl and jhonlopez@udec.cl

Table of contents

**Figure S1**. Confocal microscopy images of the mitofusin 1 protein.	S2
**Figure S2**. Effect of the incubation with **EQ-04** in the expression of the mitochondrial protein mitofusin 1.	S2<br>S2
Evaluation of the potential effect as inhibitors of the enzyme acetylcholinesterase	S3
**Table S1**. IC_{50} values for AChE and BChE for **EQ-04** and Galantamine.	S3
General Procedure for Preparation of **EQ-04**.	S3-S4
**References**	S5

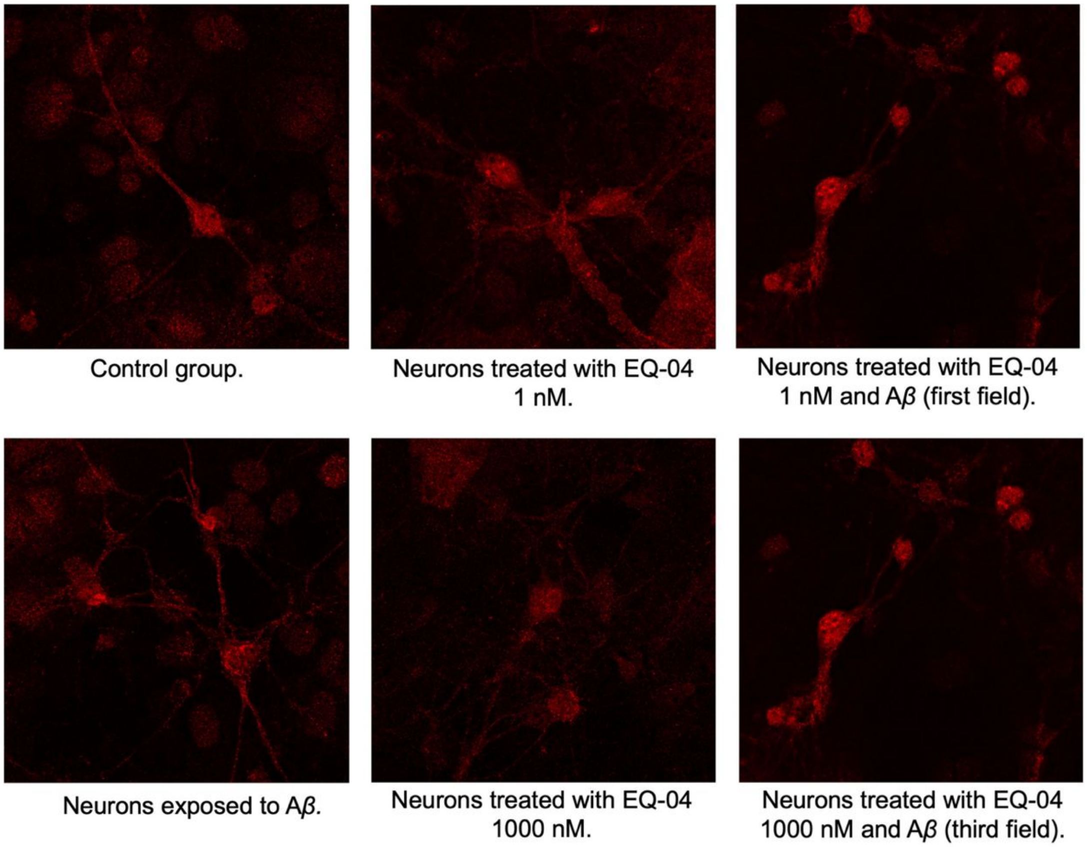

Figure S1. Confocal microscopy images of the mitofusin 1 protein.

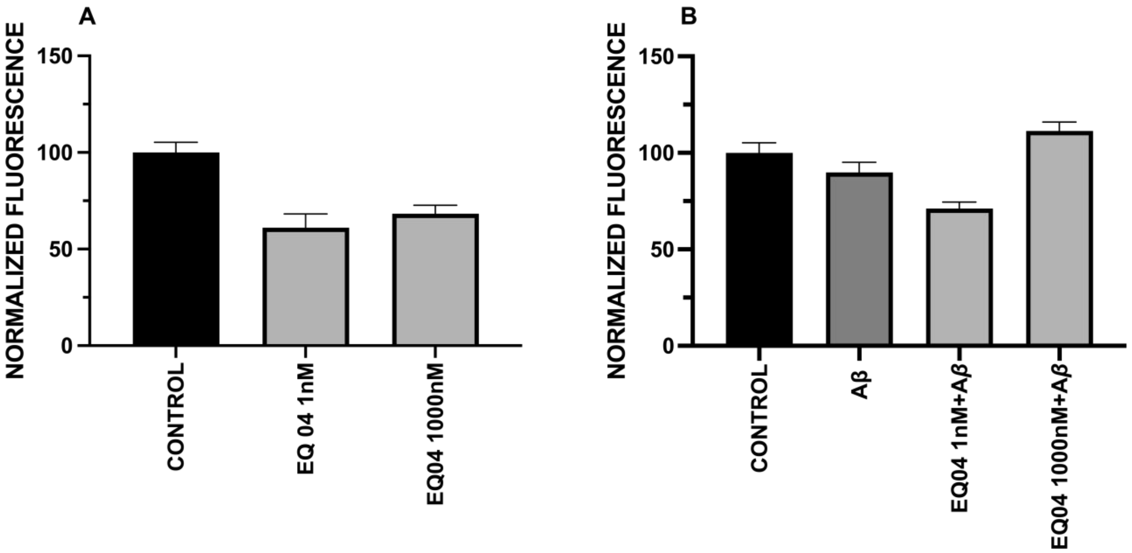

Figure S2. Effect of the incubation with **EQ-04** in the expression of the mitochondrial protein mitofusin 1.

Evaluation of the potential effect as inhibitors of the enzyme acetylcholinesterase

The evaluation of enzyme inhibition was carried out applying the Ellman's spectrophotometric method.[30] AChE (from Electrophorus electricus) and BuChE (from equine serum), 5,5′-dithio-bis-(2-nitrobenzoic acid) (DTNB), acetylthiocholine and butyrylthiocholine iodides were purchased from Sigma-Aldrich. The stock solutions of the test compounds were prepared in 100 µL of DMSO and 900 µL of phosphate buffer (8 mmol/L K_2HPO_4, 2.3 mmol/L NaH_2PO_4, 150 mmol/L NaCl, and 0.05% Tween 20 at pH 7.6). In a 96-well plate,50 µL of stock solutions were diluted with phosphate buffer to obtain concentrations between 1.95 and 1000 µg/mL of **EQ-04**. Enzyme solutions were prepared with buffer to give 0.25 units/mL and 50 µL was added to the plate. After 30 min of incubation, the substrate solution consisting of Na_2HPO_4 (40 mmol/L), acetylthiocholine/butyrylthiocholine (0.24 mmol/L) and DTNB (0.2 mmol/L) was added. The mixture was incubated for another 5 min and the absorption at 405 nm was determined with a Microtiter plate reader (Multiskan EX, Thermo). Each compound concentration was tested in triplicate. The IC_{50} values were calculated by means of regression analysis.[1]

Table S1. IC_{50} values for AChE and BChE for EQ-04 and Galantamine.

	**AChE (µM)**	**BuChE (µM)**
**EQ-04**	85.40±2.15	42.32 ±1.03
Galantamine	0.101 ±0.01	0.58±0.05

General Procedure for Preparation of EQ-04

Step 1.

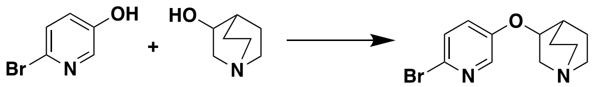

6-bromopyridin-3-ol quinuclidin-3-ol EQ-02

In a flat flask with two mouths were added the 6-bromopyridin-3-ol (1 g, 5.74 mmol), quinuclidin-3-ol (605 mg, 4.76 mmol), and 20mL of 1,4-dioxane. The mixture was allowed to stir under nitrogen, and subsequently, PPh_3 is added (1.88 g, 7.17 mmol). The reaction was cooled to 0 °C, and then the DIAD (1.41mL, 7.17 mmol in 10mL of 1,4-dioxane) solution is

slowly added. The mixture was refluxed for 48 h under nitrogen with stirring. After this time, the reaction mixture was concentrated under reduced pressure and the product was purified by chromatography column, using a gradient of dichloromethane− methanol 1:4, to give corresponding compound (white solid; 890 mg, 65%)

Step 2.

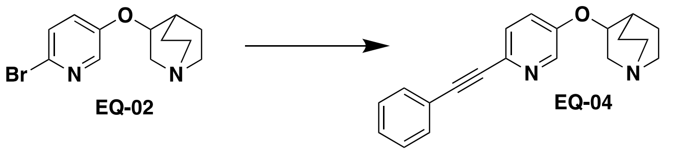

In a round bottom flask of 50 mL were added EQ-02 (500 mg, 1.76 mmol), 10mL DME-H_2O (1:1), Pd/C 10% (53 mg; 0.5 mmol), CuI (I) (53 mg, 0.28 mmol), PPh_3 (53 mg, 0.5 mmol) and K_2CO_3 (608 mg, 4.4 mmol). Then the mixture was stirring for 0.5 hours at room temperature. After this time, of the Phenylethylene (241.6 μL, 2.2 mmol) was added and the mixture was refluxed for 72 hours with stirring. The resulting suspension was filtered over celite and under vacuum. The solvent removed under reduced pressure on a rotary evaporator. Finally, the compound was purified by column chromatography, using a gradient of dichloromethane-methanol 1:4, to give corresponding compound (yellow solid; 485 mg, 90%).

References

1. Ellman, G.L.; Courtney, K.D.; Valentino, A.; Featherstone, R.M. New and rapid colorimetric determination of acetylcholinesterase activity. *Biochem. Pharmacol*. **1961**, 7(2), 88−95.
